# Effect of Postbiotic *Bifidobacterium longum* CECT 7347 on Gastrointestinal Symptoms, Serum Biochemistry, and Intestinal Microbiota in Healthy Adults: A Randomised, Parallel, Double-Blind, Placebo-Controlled Pilot Study

**DOI:** 10.3390/nu16223952

**Published:** 2024-11-19

**Authors:** Malwina Naghibi, Adria Pont-Beltran, Araceli Lamelas, Laura Llobregat, Juan F. Martinez-Blanch, Antonia Rojas, Beatriz Álvarez, Bricia López Plaza, Lucia Arcos Castellanos, Empar Chenoll, Vineetha Vijayakumar, Richard Day

**Affiliations:** 1 Medical Department, ADM Health & Wellness, London SE1 7NT, UK; 2 ADM Research and Development Center-Valencia, ADM Health & Wellness, Parc Científic Universitat de València, 46980 València, Spain; 3 Food, Nutrition and Health Platform, Hospital La Paz Institzonulute for Health Research (IdiPAZ), 28046 Madrid, Spain; 4 Medicine Department, Faculty of Medicine, Complutense University of Madrid, Plaza de Ramón y Cajal, s/n, 28040 Madrid, Spain

**Keywords:** postbiotics, gut health, microbiome, SCFAs, butyrate, healthy population, inflammation, calprotectin, abundance, *Faecalibacterium*, *Anaerobutyricum*, *Phocaeicola*, *Anaerostripes*, *Blautia*

## Abstract

Objectives: A randomised, double-blind, placebo-controlled pilot trial was conducted to assess the effect of heat-treated *Bifidobacterium longum* CECT 7347 (HT-ES1) in healthy adults with mild to moderate digestive symptoms. A total of 60 participants were recruited and received either HT-ES1 or an identical placebo for 8 weeks with a further follow-up at week 10. Methods: This study monitored changes in the total Gastrointestinal Symptom Rating Scale for IBS score (GSRS-IBS), Irritable Bowel Syndrome Symptom Severity Scale (IBS-SSS), IBS Quality of Life index (IBS-QoL), gut microbiome using 16S rRNA sequencing, and the Visceral Sensitivity Index, as well as a range of biochemical markers, anthropometric parameters, and adverse events. Results: While minimal changes were observed in gastrointestinal (GI) symptoms, the HT-ES1 group showed a significant decrease in total and non-HDL cholesterol compared to the placebo. The intervention group also exhibited a significant increase in the abundance of the genera *Faecalibacterium* and *Anaerobutyricum*, both of which were positively correlated with butyrate concentrations. Faecal calprotectin significantly increased over time in the placebo group but remained stable in the HT-ES1 group. Conclusions: Overall, these findings suggest that HT-ES1 may promote gut health by increasing butyrate-producing bacteria in the gut, maintaining normal levels of faecal calprotectin and reducing serum cholesterol.

## 1. Introduction

In recent years, there have been many developments in the research of functional foods and non-pharmacological bioactives for digestive health. Much of this research has been conducted in the microbiome space, including prebiotics, probiotics, and—more recently—postbiotics. This increase in research may be in part driven by the high prevalence of mild gastrointestinal (GI) symptoms experienced by the general population [1]. GI symptoms in otherwise healthy adults can have important consequences on overall well-being and quality of life. In a 2018 study, out of a total of 71,812 Americans, 61% reported at least one GI symptom, including heartburn, abdominal pain, bloating, diarrhoea, or constipation over a one-week period [1].

The GI microbiota is the collection of all the bacteria, archaea, and eukarya inhabiting the GI tract, and evidence indicates that the composition of the intestinal microbiota in those who have GI symptoms may be altered compared to symptom-free individuals [2]. Furthermore, it is known that a number of digestive disorders are characterised by imbalances in the composition of the *Bifidobacterium* genus [3,4]. Probiotics are defined as live microorganisms that, when administered in adequate amounts, confer a health benefit to the host [5]. Probiotics, including those belonging to the *Lactobacillus* and *Bifidobacterium* genera, have been indicated as potential candidates for the management of GI disorders such as irritable bowel syndrome (IBS) [6,7]. The genus *Bifidobacterium* has been studied extensively, and there are numerous studies indicating its potential health benefits; these include protecting against pathogenic bacteria, modulating mucosal barrier function, regulating immune and inflammatory responses, and exerting beneficial effects against conditions ranging between coeliac disease, IBS, ulcerative colitis, immunoglobulin E-associated conditions, and atopic dermatitis [8,9,10].

One strain of interest in digestive health is the breast milk isolate *Bifidobacterium longum* CECT 7347 (ES1), which has been shown in several studies to have physiological effects that may be beneficial for digestive health. In a paediatric clinical trial, children with newly diagnosed coeliac disease experienced decreased peripheral lymphocytes and decreased levels of TNF-α following supplementation with ES1 [11]. Additionally, a pilot study in a non-coeliac gluten-sensitive population demonstrated that a combination of diet and ES1 supplementation improved intestinal and extra-intestinal symptoms [12]. Notably, the beneficial probiotic bacterial–host interactions have been shown to be mediated by extracellular probiotic compounds, including organic acids, antimicrobial compounds, enzymes, and components related to the cell envelope (lipoteichoic acids, exopolysaccharides, or peptidoglycan-derived molecules) [13]. These findings underscore the potential of ES1 to exert its effects in its viable probiotic form.

Thus, considering that ES1 demonstrates significant bioactivities as a probiotic, there is interest in exploring how these effects may persist in its heat-treated form, here referred to as a postbiotic [14]. The use of postbiotics has been indicated to have several advantages over the use of live probiotic cultures, including better stability, longer shelf-life, and the ability to be included in food matrices and survive extreme conditions of processing [15]. Additionally, in populations where there may be safety concerns over the use of live bacteria, postbiotics may be a preferential alternative as they have potential applications in medical and early life nutrition [16,17]. Notably, *B. longum* CECT 7347 has been shown to retain certain functionalities in its heat-treated postbiotic form (referred to hereafter as “HT-ES1”), such as reducing inflammation in intestinal cells in an in vivo model [18]. In a recent study in an IBS population, daily consumption of either live ES1 or HT-ES1 for a period of 12 weeks was associated with statistically significant and clinically meaningful improvements in IBS-SSS, IBS-QoL, abdominal pain severity, and anxiety scores compared to placebo [7].

Despite positive preliminary data on the potential of postbiotics to improve GI symptoms, to our knowledge, the effects of postbiotic supplementation on the gut microbiome of healthy populations without any formal diagnosis of a GI disorder are yet to be explored. Here, we aim to evaluate the ability of an HT-ES1 to modulate gut microbiome composition as well as assess its ability to influence mild GI symptoms, serum biochemistry, and intestinal inflammation in a healthy adult population. 

## 2. Materials and Methods

### 2.1. Compliance with Ethical Standards

This study was conducted in compliance with the Declaration of Helsinki and National Ethical Guidelines for Biomedical and Health Research involving Human Participants, and the study protocol was approved by the Research Ethics Committee with Medical Products Hospital La Paz Institute for Health Research (10 November 2021, Ref: 57/458465.9/21; IdiPAZ). This study was registered with the USA National Library of Medicine clinical trials registry ClinicalTrials.gov (Identifier: NCT05367427) and run from the Clinical Nutrition and Dietetics Unit of the University Hospital La Paz, Madrid, Spain. Written informed consent was obtained from all participants before initiation of the study procedures. No formal patient and public involvement (PPI) activity was undertaken, so, as a result, participants were not involved in the trial design, the choice of outcomes, or the recruitment strategy for the trial.

### 2.2. Study Participants

**Figure 1:**
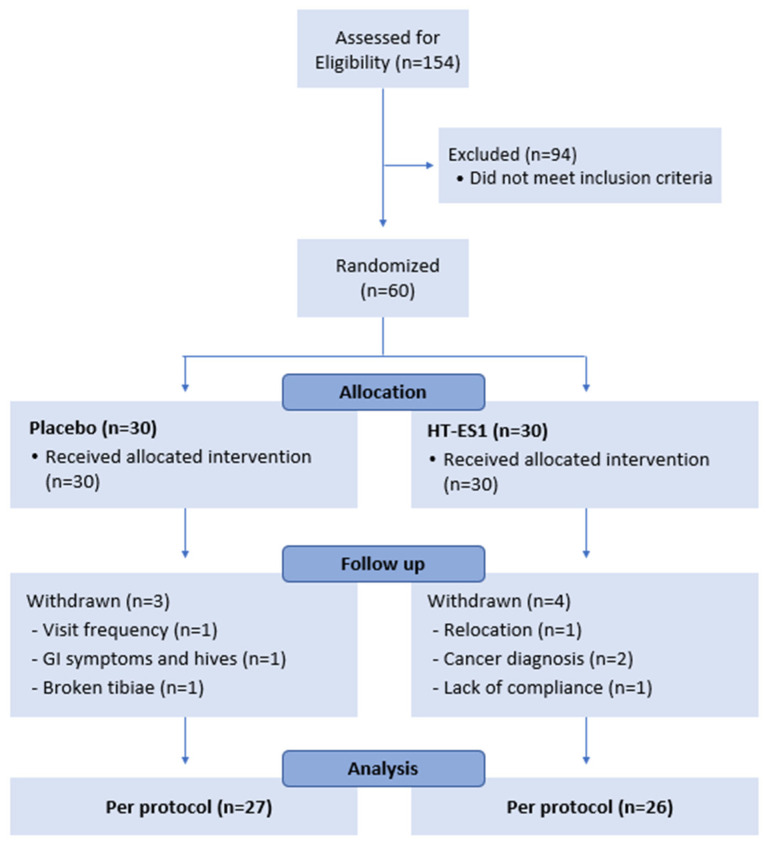
Consort flow diagram. n: number of subjects.

A total of 154 healthy volunteers were screened for eligibility; of those, 60 participants (11 males and 49 females) aged 18–65 years who scored between 13 and 39 points on the GI Symptom Rating Scale for IBS (GSRS-IBS) were included (Figure 1). More detailed inclusion and exclusion criteria are presented in Supplementary Section S1. Of the 60 randomised participants, 53 completed the study (n = 27 in placebo and n = 26 in intervention group). A total of 7 participants dropped out due to a broken tibia (n = 1), cancer diagnosis (n = 2), GI issues and hives (n = 1), due to distance, relocation, or non-compliance (n = 3). Reasons for withdrawal were determined to be unrelated to the investigational product. Therefore, the final per-protocol analysis was conducted on 53 participants.

### 2.3. Study Design and Product

This is a single-centred, placebo-controlled, pilot study to explore the safety and efficacy of the postbiotic HT-ES1 in healthy adults with mild GI symptoms. This clinical trial was carried out between February and October 2022. Participants were randomised by the Biostatistics Unit of La Paz Hospital using a 1:1 allocation ratio between the intervention group (n = 30) and the placebo group (n = 30). Participants, investigators, and assessors collecting outcome data were blinded to the assigned intervention during the study.

The intervention group was instructed to take two capsules of postbiotic HT-ES1 daily, with breakfast (2.5 × 10^9^ cells/day equivalent to 50 mg/day when prepared from a postbiotic batch at a concentration of 5  ×  10^10^ cells/g), and the placebo group was given an identical placebo (Maltodextrin) for a period of 8 weeks. HT-ES1 was prepared through a proprietary method of heat treatment of the probiotic solution followed by a drying step to create a dry powder. Capsules were produced by Korott, Alcoi, Spain. The presentation and exterior characteristics of the active and placebo were visually identical. *Bifidobacterium longum* CECT 7347 is a strain deposited in the Spanish Culture Type Collection and patented by the Spanish National Research Council.

**Figure 2:**
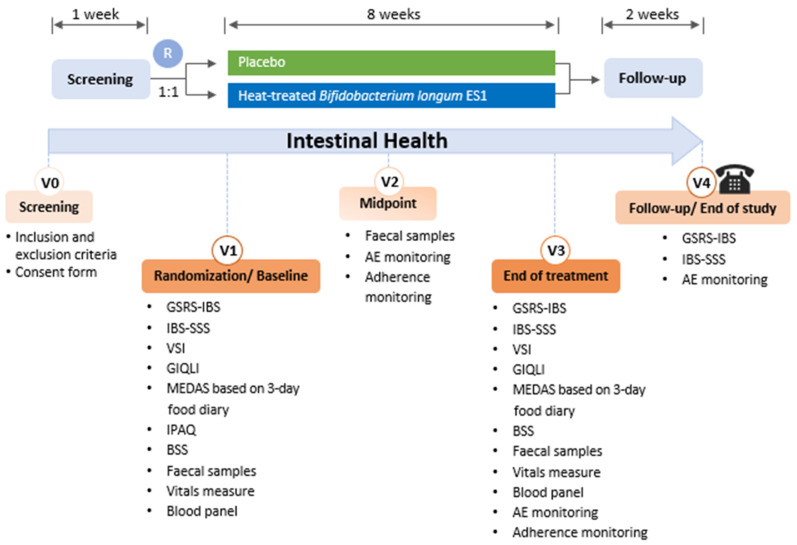
Study visit schedule. GSRS-IBS: GI Symptom Rating Scale for IBS; VSI: Visceral Sensitivity Index; IBS-SSS: Irritable Bowel Syndrome Symptom Severity Scale; GIQLI: Gastrointestinal Quality of Life Index; MEDAS: Mediterranean Diet Adherence Screener; IPAQ: International Physical Activity Questionnaire; BSS: Bristol Stool Scale; R: Randomisation.

Clinical examination and sample collection were performed at baseline and after 4 and 8 weeks of intervention. A follow-up phone call was conducted 2 weeks after cessation of the intervention (week 10) (Figure 2).

### 2.4. Outcome Measures

#### 2.4.1. Baseline and Safety Measures

The study cohort was assessed at the baseline for standard characteristics, including health habits (smoking, alcohol consumption), diet (the Mediterranean Diet Adherence Screener (MEDAS)), physical activity levels (the International Physical Activity Questionnaire (IPAQ)), and medical history. MEDAS score was also evaluated at week 8 to monitor any changes in dietary habits. Detailed methods of the assessment tools are described in the Supplementary Materials (Section S2).

Blood samples were collected at weeks 0 and 8 and analysed for full blood counts, urea and electrolytes, glucose, protein (Protein, Albumin, Prealbumin), and lipid metabolism markers (Triglycerides, HDL, LDL, non-HDL, and total cholesterol). Serum minerals (calcium, phosphorus, sodium, potassium, magnesium, chlorine) and vitamin (A, E, D, folic acid, vitamin B12) levels were also determined. Additionally, markers of inflammation (hsCRP, TNF-α, IL-4, IL-6, IL-10, and IL-12) were measured. Anthropometrics (weight, height, waist circumference, BMI) were evaluated, and bioelectrical impedance (BIA, INBODY S10) was used to assess body composition at weeks 0 and 8.

Safety variables collected at weeks 0 and 8 included blood pressure (systolic and diastolic), heart rate, and liver and kidney function (creatinine, glomerular filtration rate (GFR), alanine aminotransferase (ALT), and bilirubin).

#### 2.4.2. GI Assessment Tools

The primary outcome measure is the change in the GSRS-IBS score. The GSRS-IBS is a validated questionnaire used to assess the severity and frequency of GI symptoms related to IBS, such as abdominal pain and discomfort, bloating and flatulence, stool urgency, frequency and form, as well as satiety after meals. The GSRS-IBS includes 13 questions, and each question is rated on a Likert scale of 6 points, where 0 represents no discomfort and 6 is severe discomfort. The GSRS-IBS questionnaire was collected at weeks 0, 8, and 10 as the primary outcome of this study. Symptom severity was then classified based on the total score: mild (0–19), moderate (20–39), or severe (40–78).

As secondary outcomes, patients also completed the Irritable Bowel Syndrome Symptom Severity Scale (IBS-SSS) questionnaire at weeks 0, 8, and 10. Additionally, at the beginning and end of the intervention (weeks 0 and 8), they completed the following: the GI Quality of Life Index (GIQLI) and the Visceral Sensitivity Index (VSI) questionnaires. The Bristol Stool Form Scale (BSS) was recorded daily for 7 days prior to study visit at weeks 0 and 8. Detailed methods for the assessment tools are described in the Supplementary Materials (Section S2).

#### 2.4.3. Assessment of Faecal Samples

##### Microbiome Biochemistry

Faecal samples were collected at baseline, week 4, and week 8 using a faecal collection kit (FeelGut, Alderley edge, UK) and analysed by the ADM Research & Development Center Valencia, Spain. The concentrations of short-chain fatty acids (SCFAs) (butyric, acetic and propionic acid, and total SCFAs) in the samples were measured by high-performance liquid chromatography with refractive index detector (HPLC-RID) (Waters Corporation, Milford, MA, USA). SCFA concentration values were multiplied by 12, the dilution factor. Faecal samples were tested for calprotectin and lactoferrin using a commercially available kit (CALPRO, Abyntek Biopharma, Zamudio, Spain). The concentration of zonulin in stool was determined with an enzyme-linked immunosorbent assay (ELISA) kit (DRG Instruments GmbH, Marburg, Germany), following the manufacturer’s instructions, in Hospital La Paz Institute for Health Research (IdiPAZ).

##### Microbiome Composition

Microbial DNA was extracted from stool using the QIAsymphony PowerFecal Pro DNA Kit (Qiagen, Hilden, Germany). The V3–V4 hypervariable region of the 16S rRNA gene was amplified from genomic DNA using primers 341F (CCTACGGGNGGCWGCAG) and 805R (GACTACHVGGG TATCTAATCC). 16S-based libraries were quantified by fluorimetry using the Quant-iT™ Picofreen™ dsDNA Assay Kit (Thermofisher, Waltham, MA, USA). Libraries were pooled before sequencing on the MiSeq platform (Illumina, San Diego, CA, USA) with a 300 cycles paired reads configuration. The size and quantity of the pool were assessed on the Bioanalyzer 2100 (Agilent, Santa Clara, CA, USA) and with the Library Quantification Kit for Illumina (Kapa Biosciences, Oslo, Norway), respectively. The PhiX Control library (v3) (Illumina) was combined with the amplicon library. Image analysis, base calling, and data quality assessment were performed on the MiSeq instrument (MiSeq Control Software (MCS v2.6.2.1)). Forward and reverse sequences were merged using the BBMerge package of BBMap V.38 software [19]. The amplification primers were trimmed to reduce the bias in the annotation step using Cutadapt v 1.8.1 [20].

A quality filter was applied to delete poor-quality sequences using the Reformat package of BBMap V.38 software. Sequences lower than 200 nts were removed from the analysis. Those bases in extreme positions that did not reach Q20 or a greater Phred score were removed. Sequences whose average quality did not surpass the Q20 threshold, as a mean quality of the whole sequence, were also deleted. The reads were processed using the DADA2 algorithm ‘denoise-single’ command [21]. Error rates were learned from a set of subsampled reads using ‘learnErrors’, and the sample inference algorithm was applied with the ‘dada’ function to generate the Amplicon Sequence Variants (ASVs). The chimeric ASVs were removed using the ‘removeChimeraDenovo’ function. The taxonomy of the ASVs was annotated against the NCBI 16S rRNA database version 2023 using blastn version 2.2.29+ [22]. The taxonomy of the ASVs that had been assigned with a lower percentage identity than 97% was reassigned using the NBAYES algorithm [23] from QIIME2 platform v2021.8 [24]. The NBAYES classifier was previously trained on V3-V4 regions of the 16S rRNA gene from the SILVA v.138 database [25]. ASVs identified to a bacteria kingdom and with at least 0.05% of relative frequency in at least 5 samples were kept for the statistical analysis. Sequence files and metadata for the microbiome analyses were deposited in NCBI SRA under BioProject PRJNA1164946.

#### 2.4.4. Compliance and Adverse Event Reporting

Compliance was assessed by monitoring the number of capsules returned at each visit, and once the intervention period was over, the remaining containers were returned to the Principal Investigator. Concomitant medication and adverse events were also monitored.

### 2.5. Statistical Analysis

#### 2.5.1. Statistical Analysis of Questionnaires and Physical and Biochemical Parameters

Normally distributed data were analysed using Student’s *t*-tests, and non-parametric data were analysed using the Mann–Whitney U-test. Between-group changes from the baseline were analysed using an independent *t*-test. Within group analyses were conducted using paired sample *t*-tests. All tests were two-tailed with significance set at *p* < 0.05. Analyses were conducted on per-protocol (PP) populations using SPSS 21.0 at La Paz University Hospital’s Biostatistics Section.

The Wilcoxon test was used to find differences in calprotectin, lactoferrin, and short-chain fatty acid concentrations between times on each group and between groups on each time.

#### 2.5.2. Microbiome Data

Data were normalised using a rarefaction technique from Phyloseq R package v1.34 [26] to perform alpha diversity analysis. Shannon, Simpson, and Richness indexes were calculated using vegan R package v2.5-7 [27]. The Bray–Curtis dissimilarity matrix and permutational analysis of variance (PERMANOVA) analysis for beta diversity were performed using vegan R package v2.5-7 after normalisation by relative frequency for each sample. PERMANOVA was used to assess the differences in the microbiome composition among groups, times, and subjects and was also used to evaluate the influence of the MEDAS index on the microbiome.

Differential taxa abundance was analysed using the DESeq2 R package v.1.30.1 [28]. The normalisation was based on the ‘Relative Log Expression’ method. The ‘EstimateSizeFactors’ function was used to calculate the scaling factors using the median ratio between taxa abundances and the geometric mean. We used the ‘PosCounts’ method, which deals with taxa that have multiple zeros in most of the samples, as usually occurs in metagenomics. A taxon was considered differentially abundant with a Benjamani–Hochberg (“BH”) multiple testing correction adjusted *p*-value < 0.05 and if it was present in at least 50% of the samples of one of the compared groups. Heatmaps were constructed using ComplexHeatmap R package v.2.11.1 [29]. For this analysis, only subjects with three samples obtained thought time were considered.

MaAslin2 R package v1.4 [30] was used to study correlations between microbial abundances and organic compounds. A linear model test was performed for each variable, with the variable as the fixed effect. The microbial taxa counts were normalised using the DESeq2 normalisation method, and the normalised counts were log-transformed. Only taxa present in more than 10% of the samples were considered.

The Wilcoxon test was used to find differences in alpha diversity metrics between times on each group and between groups on each time. It was also used to find differences in the intra-subject over-time changes between the groups on certain taxa abundances.

## 3. Results

### 3.1. Baseline Characteristics of Participants

The baseline characteristics of the enrolled subjects were comparable (Table S1A–C). The IPAQ showed no significant differences between groups at the baseline, with 73.3% of the population reporting moderate physical activity, 10% stating a predominantly sedentary lifestyle, and 16.7% of the cohort reporting high physical activity. Regarding dietary habits, the MEDAS index indicated that the placebo group followed a more Mediterranean-style diet compared to the HT-ES1 group at the baseline [placebo: 8.0 ± 1.2 vs. HT-ES1: 7.1 ± 1.4, *p* = 0.007]. However, it is important to note that the mean score for neither group reached the nine-point threshold, which has been previously established as the cut-off for a diet that meets the recommendations of the Mediterranean diet [31].

### 3.2. Biochemical Outcomes and Safety Parameters

The blood biochemical assessment over the study duration indicated that there was a significant between-group difference in the change in total cholesterol, [HT-ES1: −12.83 ± 46 vs. placebo: +8.04 ± 22.42 (*p* = 0.01)] and non-HDL cholesterol [HT-ES1: −0.85 ± 17.06 vs. placebo: +8.3 ± 19.32 (*p* = 0.044)] at the end of the intervention (Table 1 and Table S2).

Significant within-group changes were observed in LDL cholesterol, albumin, ferritin, vitamin D, vitamin E, fat mass, urate, bilirubin, and systolic blood pressure (SBP) in both groups, or specifically in either the placebo or probiotic group (Table 1, Tables S2 and S3). Of note, during the course of the study, three participants (two in HT-ES1 group and one in placebo) were given vitamin D supplements due to deficiencies.

Safety variables such as blood pressure (systolic and diastolic), heart rate, and kidney and liver function (creatinine, GFR, ALT, and bilirubin levels) were comparable between the groups throughout the intervention (Table S3).

### 3.3. Adverse Event

Overall, 27 participants (15 in HT-ES1, 12 in placebo) reported a total of 39 adverse events (AEs). Only two of the AEs were classified as related to the consumption of the investigational product (gastralgia and foul gases), and three others (nausea, bloating, and reflux) were deemed to be possibly related to the postbiotic. In the placebo group, three AEs (nausea, diarrhoea, foul gases) were suspected to be related to the study product. There were no SAEs related to the investigational product reported.

### 3.4. GI Assessment

No significant between-group differences were observed for the total GSRS-IBS, IBS-SSS, GIQLI, VSI, or BSS scores after 8 weeks of intervention (Table 2).

Some outcomes exhibited a significant within-group decrease at the 10-week follow-up. The diarrhoea subscale of the GSRS showed a significant within-group decrease in both groups [HT-ES1 2.03 ± 0.65 vs. 1.49 ± 1.09 *p* < 0.01 and placebo 2.2 ± 0.91 vs. 1.52 ± 1.13 *p* < 0.05]. Additionally, a significant within-group reduction in the total GSRS-IBS score was observed at the follow-up in both groups [HT-ES1 27.08 ± 8.32 vs. 21.92 ± 14.1 *p* < 0.05 and placebo 28.62 ± 9.25 vs. 22.35 ± 14.19 *p* < 0.05] (Table S4). The placebo group also showed a significant within-group decrease in total IBS-SSS scores [187.88 ± 93 vs. 163.65 ± 80.41, *p* < 0.05]. However, despite being statistically significant, this change is not considered clinically meaningful, and baseline values, although not significantly different, reflected different severity categories between groups.

### 3.5. Faecal Health Biomarkers

#### 3.5.1. Inflammation Markers

**Figure 3:**
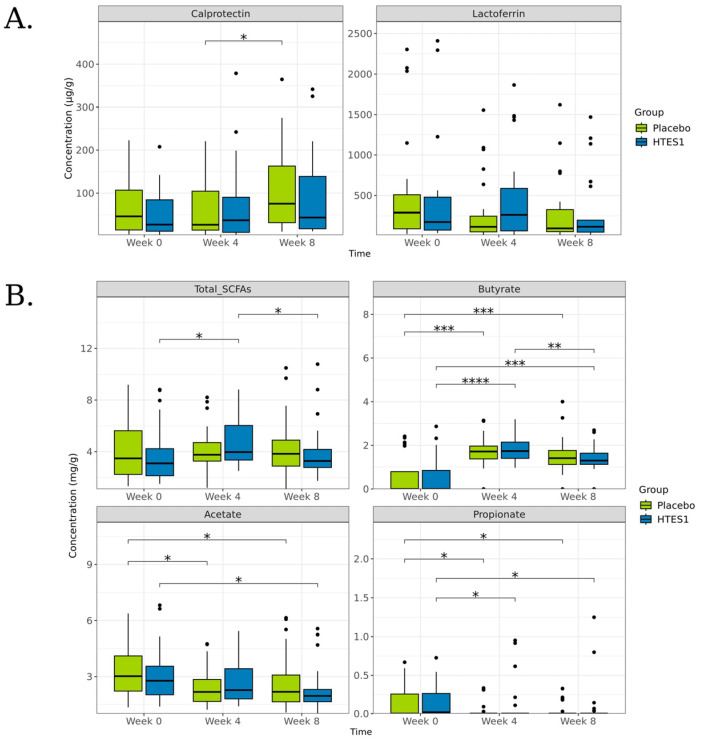
(**A**) Boxplots on calprotectin and lactoferrin concentrations and (**B**) organic compound concentrations in each group over time; weeks 0, 4, and 8. The Wilcox test was applied between times in each group and between groups on each time. * *p* < 0.05, ** *p* < 0.01, *** *p* < 0.001, **** *p* < 0.0001.

Calprotectin and lactoferrin were measured in faecal samples at the baseline, week 4, and week 8. There was no significant difference in the faecal calprotectin concentration between the intervention and placebo groups at the end of the study. However, in the placebo arm, a significant increase in faecal calprotectin levels between weeks 4 and 8 of the intervention period was observed [wk4: 26.84 ± 59.84 vs. wk8: 75.40 ± 95.04 *p* = 0.039] (Figure 3A). No within-group changes were observed in the HT-ES1 group. The lactoferrin concentration did not vary significantly over time in either group.

#### 3.5.2. Short-Chain Fatty Acids

No significant differences in SCFAs were observed between groups at any time point (Figure 3B). A significant within-group change in total SCFA was observed in the HT-ES1 arm from week 0 to week 4 [3.09 ± 2.15 vs. 3.97 ± 1.77 (*p* = 0.027)], and there was a significant decrease from week 4 to 8 [3.97 ± 1.77 vs. 3.27 ± 2.07 (*p* = 0.031)]. In contrast, no significant changes in the total SCFA concentration were observed over time in the placebo group.

While analysing individual SCFAs, within-group changes were observed in both groups for butyric acid, propionic acid, and acetic acid (Figure 3B). A significant increase in the butyric acid concentration was noted in both groups over time [placebo: wk0: 0 ± 0.88 vs. wk4: 1.72 ± 0.64 (*p* = 0.0002) and vs. wk8: 1.41 ± 0.78 (*p* = 0.0004) and HT-ES1 group: wk0: 0 ± 0.88 vs. wk4: 1.74 ± 0.61 (*p* = 0.000004) and vs. wk8: 1.30 ± 0.56 (*p* = 0.0002)]. A significant decrease in the propionic and acetic acid concentration was observed in both groups over time: propionic acid [placebo: wk0: 0 ± 0.21 vs. wk4: 0 ± 0.08 (*p* = 0.025) and vs. wk8: 0 ± 0.09 (*p* = 0.045) and HT-ES1 group: wk0: 0.02 ± 0.21 vs. wk4: 0 ± 0.27 (*p* = 0.05) and vs. wk8: 0 ± 0.28 (*p* = 0.037)]; acetic acid [placebo: wk0: 3.02 ± 1.38 vs. wk8: 2.19 ± 1.49 (*p* = 0.05), and HT-ES1: wk0: 2.78 ± 1.42 vs. wk8: 1.97 ± 1.17 (*p* = 0.011)] (Figure 3B).

### 3.6. Microbiome Composition

#### 3.6.1. Composition of Gut Microbiome

A total of 947 ASVs were taxonomically identified as bacteria and passed the prevalence filter. In total, 82.58% of these ASV were classified at the genus level. Overall, the study population had a microbiome mainly dominated by the genera *Phocaeicola* (10.31 ± 5.46%), *Faecalibacterium* (7.68 ± 3.52%), *Bacteroides* (6.49 ± 3.98%), *Blautia* (6.46 ± 3.55%), and *Bifidobacterium* (4.79 ± 4.33%) (Figure S1).

#### 3.6.2. Diversity Analysis

**Figure 4:**
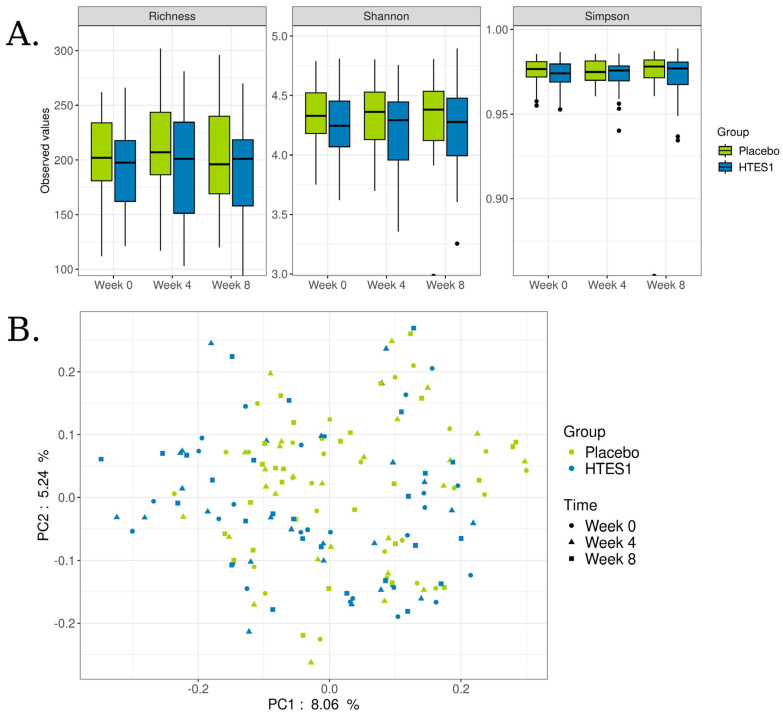
(**A**) Boxplots of Richness, Shannon, and Simpson indexes in placebo and HT-ES1 groups over weeks 0, 4, and 8. The Wilcoxon test was applied between times on each group and between groups on each time, with no significant results. (**B**) PCoA plot of beta diversity based on Bray–Curtis distance analysis in different groups and times.

No significant differences were observed in richness or alpha diversity, measured by the Shannon or Simpson indices, at either time point when comparing the HT-ES1 or placebo groups (Figure 4A). Additionally, we performed principal coordinate analysis (PCoA) using the Bray–Curtis distance to assess whether the overall gut microbiome composition differed between time points and groups. The analysis revealed no distinct clustering by either group or time (Figure 4B).

#### 3.6.3. Permutational Analysis of Variance

A PERMANOVA analysis using the Bray–Curtis distance method, and including the MEDAS index as a factor, showed that MEDAS had no significant influence on the microbiome variability [R = 0.002, (*p* = 0.7)] (Table S5A). Moreover, no significant correlation score was found between the MEDAS index and other organic compounds and clinical variables (Figure S2). The PERMANOVA analysis based on a simplified model indicated significant differences in microbiome profiles among groups [R = 0.0192, (*p* = 0.001)], time [R = 0.0084, (*p* = 0.001)], and subject [R = 0.8251, (*p* = 0.001)] (Table S5B).

#### 3.6.4. Differential Abundance of Bacterial Taxonomic Features

**Figure 5:**
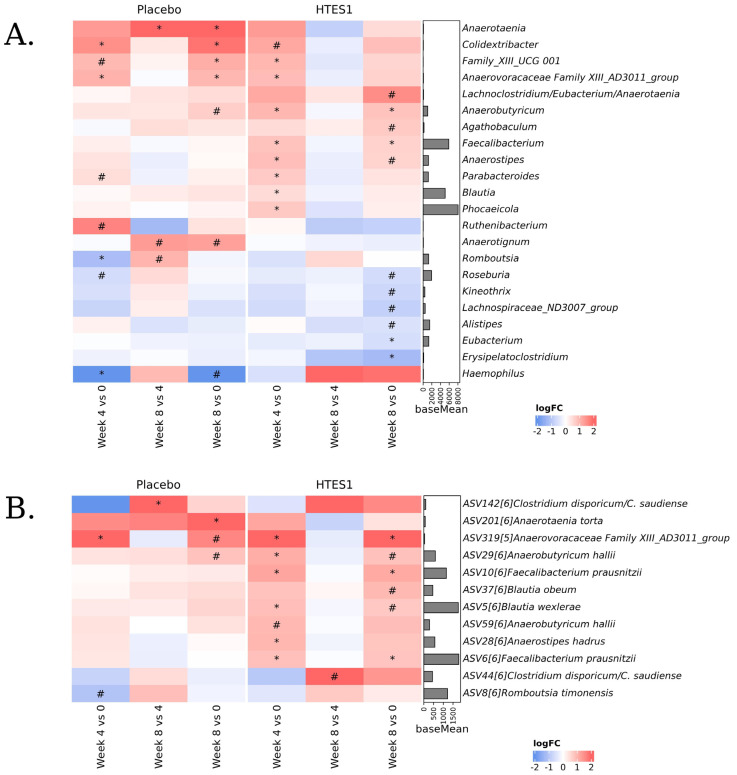
Differentially abundant taxa by DESeq2. Heatmaps showing the ‘log fold change’ (Log2FC) resulting from between time comparisons per group on (**A**) genera and (**B**) ASV abundances. Red indicates that the taxon is over-represented in the first group of the comparison, while blue indicates that the taxon is over-represented in the second group. # 0.05 < adj. *p* < 0.1, * adj. *p* < 0.05. In # and *, the presence of the taxon in at least 50% of samples of at least one of the compared groups. BaseMean bar plots show the mean abundances of each genus.

Differential abundance analysis identified a number of bacterial taxa for which abundance significantly differed over time within the placebo or HT-ES1 group. The differences in abundance were primarily observed between week 0 and 4 (Figure 5). At the genus level (Figure 5A), within the placebo group, *Anaerotaenia* experienced a significant increase between week 4 vs. 8 and week 0 vs. 8, while no significant difference was observed in the HT-ES1 group. Additionally, *Haemophilus* was shown to significantly decrease in the placebo group at week 0 vs. 4 [adj. *p* < 0.05], which did not occur in the HT-ES1 group.

A significant increase in abundance, for butyrate-producing bacteria such as *Faecalibacterium*, *Anaerobutyricum*, *Phocaeicola*, *Anaerostripes*, and *Blautia*, at week 0 vs. 4 was observed only in the HT-ES1 group [adj. *p* < 0.05]. *Faecalibacterium* and *Anaerobutyricum* abundances were also significantly increased between week 0 and 8 [adj. *p* < 0.05] in the HT-ES1 group. Of note, *Anaerobutyricum* showed a non-significant increase in the placebo group. In the HT-ES1 arm, a significant decrease in *Eubacterium* and *Erysipelatclostridium* was observed at week 8 vs. 0.

The ASV annotated as *Faecalibacterium prausnitzii* (ASV10 and ASV6), *Anaerobutyricum hallii* (ASV29), *Anaerostripes hadrus* (ASV28), and *Blautia wexlerae* (ASV5) had a significant abundance increase at week 0 vs. 4 in the HT-ES1 group, which was not observed in the placebo group at the same time interval (Figure 5B). The abundance of *Faecalibacterium prausnitzii* remained significantly increased at week 0 vs. 8, while the abundance of *A. hallii*, *B. obeum* (ASV37), and *B. wexlerae* had a trending increase at week 0 vs. 8 (Figure 5B).

**Figure 6:**
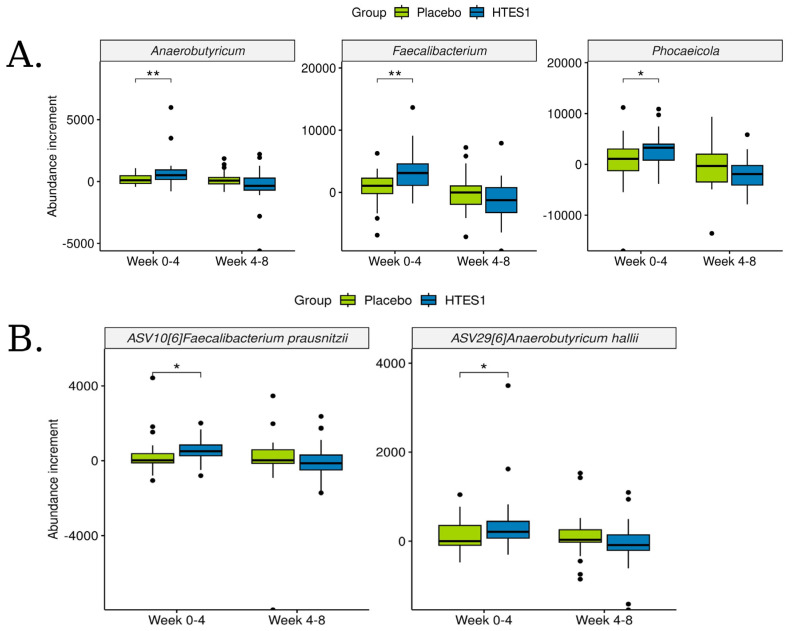
Boxplots on intra-subject differences between weeks 0–4 and weeks 4–8 times on each group of (**A**) genera and (**B**) ASV abundances normalised by DESeq2. Wilcox test was applied between groups on each period. * *p* < 0.05, ** *p* < 0.01.

The genera identified as significantly abundant by DESeq2 were further analysed, examining the differences per subject during intervals of weeks 0–4 and 4–8. To evaluate specific abundance increases, the Wilcoxon test was performed comparing the placebo and HT-ES1 groups for each period. *Faecalibacterium*, *Phocaeicola*, and *Anaerobutyricum* were found to be significantly increased in the HT-ES1 group compared to the placebo group at weeks 0–4 (Figure 6A). In all cases, variations in the increments were shown only in the first period of study (weeks 0–4). When the same analysis was applied at the ASV level, *F. prausnitzii* and *A. hallii* were shown to have a significant abundance increment [*p* < 0.05] in the HT-ES1 vs. placebo group and, as in the case of the genus level, they remained stable at weeks 4–8 (Figure 6B).

#### 3.6.5. Correlation Analysis

**Figure 7:**
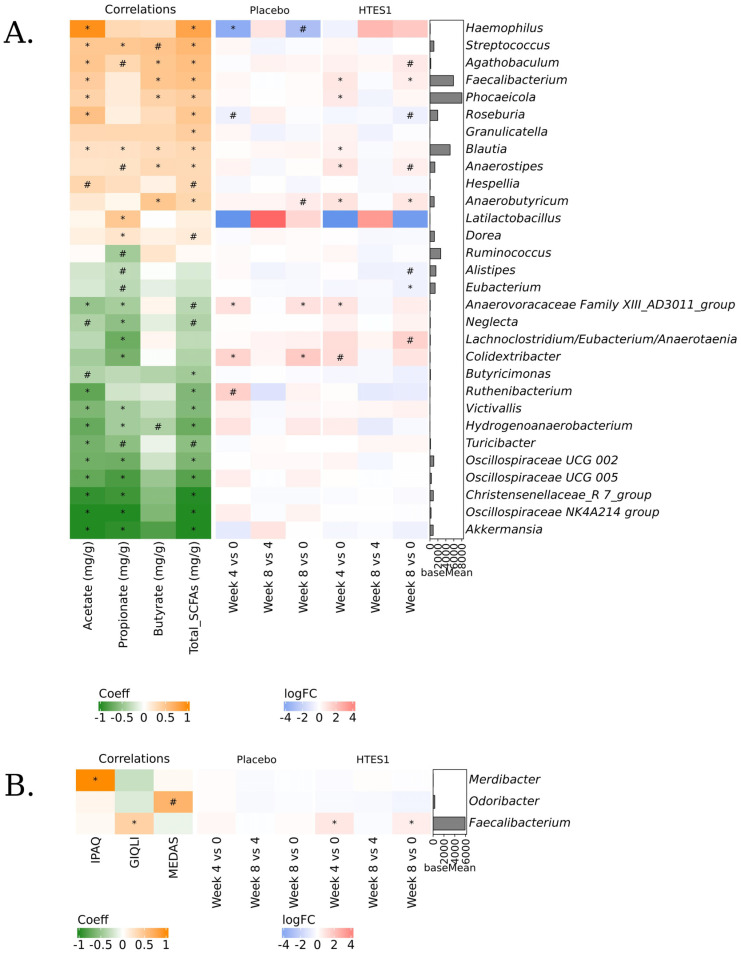
Correlations: heatmaps showing the Maaslin2 Coefficient (Coeff) of correlations between (**A**) organic compound concentrations and genera abundances and (**B**) questionnaires and genera abundances. # 0.05 < adj. *p* < 0.1, * adj. *p* < 0.05. Orange colour means it is directly correlated, while green colour means inversely correlated. Red colour means that the taxon is over-represented in the first group of the comparison, while blue colour means that the taxon is over-represented in the second group. # 0.05 < adj. *p* < 0.1, * adj. *p* < 0.05. In # and *, the presence of the taxon occurs in at least 50% of samples of at least one of the compared groups. BaseMean bar plots show the mean abundances of each genus.

A linear mixed-effects model using the Maaslin2 tool was implemented to determine if correlations between genera and levels of SCFAs, butyric acid and acetic acid, and the clinical questionnaires existed. The genera *Agathobaculum*, *Faecalibacterium*, *Blautia*, *Anaerostripes*, *Phocaeicola*, and *Anaerobutyricum* positively correlated with butyric acid concentration, while *Haemophilus* abundance correlated with acetic acid content (Figure 7A). At the ASVs level, butyrate-producing species *F. prausnitzii*, *A. hallii*, *B. wexlerae*, *B. luti*, *A. hadrus*, and *Anaerovoracaceae* family XIII_AD3011_group had a positive correlation with butyric content [*p* < 0.05] (Figure S3). *Faecalibacterium* genus abundance positively correlated with the GIQLI, with higher scores indicating better gut health (Figure 7B).

## 4. Discussion

This study investigated the potential of postbiotic HT-ES1 to modulate the intestinal microbiota, influence clinical presentation, and impact a range of commonly assessed haematological and biochemical parameters in healthy adults experiencing mild GI symptoms. Our results demonstrate that 8 weeks of HT-ES1 intake significantly reduced total and non-HDL cholesterol levels. The postbiotic notably increased butyrate-producing bacteria while maintaining stable calprotectin levels. However, HT-ES1 did not outperform the placebo when comparing scores from commonly used GI ratings tools in this population of healthy individuals with occasional digestive symptoms. The incidence of AEs in this study was low, with only two AEs attributed to HT-ES1 and three being potentially related. All AEs were considered mild and resolved quickly. There were no significant differences between groups in AEs reporting or in the safety parameters measured (vital signs, liver function, kidney function), adding to the safety profile of the postbiotic.

An increase in butyrate-producing bacteria is frequently considered to be desirable as butyrate acts as an energy source for colonocytes, plays an integral role in the maintenance of colonic health [32], and is therefore considered a reference indicator when screening for “next-generation probiotics” [33]. Additionally, butyrate has been shown to influence cell proliferation and differentiation, iron absorption, intestinal motility and barrier function, oxidative stress, immune regulation, and cholesterol synthesis [34]. In this study, butyric acid producers, belonging to the phylum Firmicutes: *Faecalibacterium*, *Anaerobutyricum*, *Phocaeicola*, *Anaerostripes*, and *Blautia*, showed a significant increase in the HT-ES1 group which was not observed in the placebo group. Furthermore, correlation analysis also revealed that the genera *Faecalibacterium*, *Anaerobutyricum*, *Phocaeicola*, *Anaerostripes*, and *Blautia* positively correlated with butyric acid concentrations. Species belonging to these genera have been associated with a variety of health benefits. The *Faecalibacterium* genus in particular has been identified as having promising health associations [35], while its reduced abundance has been associated with various conditions, including inflammatory bowel diseases (IBD), Alzheimer’s disease, and post-acute COVID-19 syndrome (long COVID) [36,37,38]. Thus, finding ways to increase the abundance of this health-associated genus is highly desirable. Notably, the genus *Faecalibacterium* belongs to the *Oscillospiraceae* family, which also observed a significant within-group increase between weeks 0 and 4 and a trend increase between weeks 0 and 8 in the HT-ES1 group. Sequencing data and metabolic profiling suggest that members of the *Oscillospiraceae* family, such as *Oscillospira*, may have the ability to produce SCFAs such as butyrate [33]. *Anaerobutyricum*, a known butyrate producer [39] and species belonging to this genus (*A. halii*), has recently been termed a next-generation probiotic as a result of its associated bioactive properties [40]. The *Phocaeicola* genus, which belongs to the family *Bacteroidaceae* (phylum Bacteroidetes), has been indicated to play a critical role in gut health [41], contributing not only to the degradation of complex heteropolysaccharides but also vitamin synthesis [42]. In recent years, several studies have indicated that *Blautia* may play a role in the aetiology of certain inflammatory and neoplastic conditions; its abundance has been shown to be significantly reduced in patients with Crohn’s disease and colorectal cancer [43]. This is in contrast to earlier studies that showed a higher abundance of *Blautia* in subjects with IBS and ulcerative colitis [43]. This may indicate that health effects may be exhibited by specific species or even strains rather than the whole genus, highlighting the importance of analysing the microbiome at the species level to obtain a holistic insight into the specific bioactivities associated with different bacterial species.

Considering this, we conducted ASV-level microbiome analysis, identifying an increased abundance of specific butyric acid-producing species: *Faecalibacterium prausnitzii*, *Anaerobutyricum hallii*, *Anaerostripes hadrus*, and *Blautia wexlerae* [44,45,46,47]. Notably, levels of butyrate increased from week 0 to 4 in both groups. In the HT-ES1 group, this increase can be attributed to the increase in butyrate producers *Faecalibacterium*, *Anaerobutyricum*, *Phocaeicola*, *Anaerostripes*, and *Blautia*, which were positively correlated with butyric acid concentrations. The non-significant increase in butyrate-producing *Anaerobutyricum*, genera observed in the placebo arm, offers an explanation for why butyrate production remained similar in both groups. An interesting observation in this study was that acetate production was decreased in both groups following treatment. Indigenous intestinal species from the genera *Faecalibacterium (F. prausnitzii)*, *Anaerostipes*, *Eubacterium*, and *Roseburia* can utilise cross-feeding pathways, converting acetate and lactate to butyrate [10]. In fact, acetate is a major driver of butyrate production by members of the *Faecalibacterium* genus [48], and in in vitro assays, culture medium supplemented with acetate has been shown to increase the growth of *Faecalibacterium* [49,50,51].

Butyric acid-producing bacteria play important roles in maintaining gastrointestinal homeostasis. *B. wexlerae* has been linked to reducing inflammation associated with obesity-related complications, and thus methods to increase its levels offer potential microbiota-based strategies for supporting overall health [52]. Similarly, *F. prausnitzii* has been shown to inhibit NF-kB expression and IL-8 secretion in the context of colitis, reducing inflammation while in parallel preventing pathogen colonisation [36,53]. During our 8-week study, there were no significant changes in serum anti-inflammatory (IL-4, IL-10) or pro-inflammatory parameters (IL-6, IL-12, TNF-α). However, faecal calprotectin, which is a marker of inflammation in the intestine [54,55] and a surrogate marker of IBD [56], was increased in the placebo group but remained stable in the HT-ES1 group. While these findings suggest that HT-ES1 may have a stabilising effect on intestinal inflammation, only mild changes in symptoms were observed in this population. The validated assessment tools used to measure GI symptoms (IBS-SSS and GSRS-IBS) are designed to measure changes in individuals with a clinical diagnosis of IBS. Our previous publication involving IBS-D predominant individuals demonstrated a clinically meaningful improvement in GI symptoms following HT-ES1 intake [7].

Notably, a significant difference in the change in total cholesterol and non-HDL cholesterol in the HT-ES1 group in comparison to the placebo was observed. Preclinical and clinical studies have indicated that probiotic supplementation may have a beneficial effect on serum lipid profiles [57,58]. Meta-analysis of 32 randomised controlled trials (RCTs) involving 1971 patients demonstrated that probiotics may reduce total cholesterol (TC), and specific species such as *L. acidophilus* and *Bifidobacterium lactis* were shown to significantly reduce serum TC [59].

In the HT-ES1 group, a significant increase in vitamin D level from the baseline was observed. However, during data analysis, it was noted that three subjects were supplemented for their vitamin D deficiency over the duration of the intervention (two in the HT-ES1 and one in the placebo group). Furthermore, this clinical trial was carried out between February to October, and it is possible that seasonal variation in vitamin D occurred. To establish the role of HT-ES1 in vitamin D homeostasis, future studies should take into account the possibility of seasonal variation as well as supplementation. Indeed, a recent systematic review and meta-analysis of 308 studies with 7,947,359 participants found that 15.7%, 47.9%, and 76.6% of participants had serum 25-hydroxyvitamin D levels less than 12, 20, and 30 ng/mL, respectively [60], with normal ranges falling between 30 and 100 ng/mL. Interestingly, evidence indicated that combined supplementation of vitamin D and probiotics may have superior bioactivity in modulating the intestinal microbiome and metabolome [61,62,63]. Furthermore, probiotics have been shown to increase the intestinal absorption of vitamin D [62].

Although our study brings new insights into the effects of HT-ES1 on a generally healthy population with mild GI symptoms, we acknowledge that this clinical study had some limitations. Firstly, as this was a pilot study, no formal sample size calculation was performed. This might have led to the study population being underpowered, limiting the ability to detect differences between groups. Secondly, the population was selected from a healthy cohort with mild symptoms, which may have contributed to the absence of statistically significant changes in the primary outcome and the scores from the other GI assessment tools. Thirdly, because this was a feasibility study, some parameters such as supplement intake were not considered as exclusion criteria, and this may have affected some of the outcomes measured.

Despite its limitations, our study has several strengths. It is the first to evaluate the effects of HT-ES1 on a generally healthy population with mild GI symptoms. In addition to the previously published trial using HT-ES1, the results from this trial further contribute to the valuable safety data on postbiotics and specifically on HT-ES1. HT-ES1 demonstrated an interesting modulatory effect on the GI microbiome of this cohort, as evidenced by increased levels of beneficial butyrate-producing genera (*Faecalibacterium*, *Anaerobutyricum*, *Phocaeicola*, *Anaerostripes*, *and Blautia*) and species (*F. prausnitzii*, *A. hallii*, *A. hadrus*, and *B. wexlerae*) in the GI tract. This microbiome-modulatory effect is the first time such data have been published for this strain and represents one of the few published studies to date which show the microbiome-modulating effects of a postbiotic on a healthy adult population. Additionally, interesting effects were observed on cholesterol, vitamin D, and calprotectin levels.

Additional research will help to further define the potential of the postbiotic HT-ES1, especially examining the mechanisms behind HT-ES1′s microbiome modulation. The increase in *Faecalibacterium* and *Anaerobutyricum* from HT-ES1 supplementation may stem from cross-feeding interactions, where metabolites produced by *B. longum* (like lactate and acetate) support the growth of these butyrate-producing bacteria [64]. In vitro experiments and animal models indicate that *Bifidobacterium longum* has anti-inflammatory effects on intestinal cells [65], thereby creating a favourable environment for these beneficial microbes. Studies focusing on metabolite profiling could clarify the pathways through which HT-ES1 enhances the abundance of these butyrate-producing genera. Further larger and focused studies could provide valuable insights into HT-ES1′s impact on inflammation markers, cholesterol levels, and sustained microbiome changes, thereby contributing to the promotion of overall digestive and gut health.

## 5. Conclusions

The results of this randomised, double-blind, placebo-controlled pilot trial demonstrate that the consumption of HT-ES1 for 8 weeks increased the abundance of butyrate-producing bacteria, a key source of bacterial SCFAs essential for intestinal health. Furthermore, the calprotectin levels remained stable in the HT-ES1 group, while a significant increase was noted in the placebo group between week 4 and 8, suggesting a potential protective effect of the postbiotic against intestinal inflammation. Interestingly, a significant decrease in total cholesterol from baseline to week 8 was noted in the HT-ES1 group compared to placebo. Adverse event reporting demonstrated that the postbiotic was safe for use, as the observed AEs were consistent with expectations and aligned with those reported in published trials of similar products. Overall, these findings suggest that HT-ES1 is effective in promoting beneficial gut microbiota and maintaining intestinal health while demonstrating a favourable safety profile.

## Figures and Tables

**Table 1:** Per-protocol analysis of changes in serum biochemistry and faecal parameters.

	HT-ES1		Control		
	n = 26		n = 27		
	Week 0	Week 8	Week 0	Week 8	*p*-Value #
**Serum Biochemistry**					
Glucose (mg/dL)	92.85 ± 6.69	90.62 ± 7.27	91.7 ± 5.92	91.52 ± 8.34	ns
Cholesterol (mg/dL)	193 ± 29.18	180.17 ± 48.72	191.63 ± 28.79	199.67 ± 33.95	0.01
HDL (mg/dL)	57.88 ± 13.01	55.46 ± 13.4	64.44 ± 15.03	64.15 ± 14.69	ns
Non-HDL (mg/dL)	135 ± 26.51	134.15 ± 29.87	127.19 ± 30.45	135.48 ± 37.44 *	0.044
LDL (mg/dL)	118.32 ± 23.29	128.92 ± 31.05 **	110.3 ± 27.09	127.48 ± 37.43 ***	ns
Triglycerides (mg/dL)	84.12 ± 46.61	93.77 ± 64.44	86.07 ± 39.71	91.85 ± 51.69	ns
Total proteins (g/dL)	7.16 ± 0.33	7.06 ± 0.37	7.24 ± 0.62	7.07 ± 0.56 *	ns
Albumin (mg/dL)	4.58 ± 0.21	4.32 ± 0.31 ***	4.56 ± 0.32	4.35 ± 0.33 **	ns
Prealbumin (mg/dL)	24.51 ± 4.58	25.06 ± 4.55	25.86 ± 3.44	27.04 ± 4.26	ns
Ferritin (ng/mL)	77.38 ± 75.26	79.62 ± 66.93	51.04 ± 47.02	61.48 ± 43.53 ***	ns
**Faecal parameters**					
Zonulin (ng/mg)	177.38 ± 127.69	208.87 ± 137.89	185.69 ± 98.37	241.31 ± 222.74	ns

# Change of HT-ES1 vs. placebo. Intragroup change from baseline: * *p* < 0.05, ** *p* < 0.01, *** *p* < 0.001. ns: non-significant.

**Table 2:** Health questionnaire total scores.

Questionnaire	HT-ES1	Placebo	*p*-Value #
	(n = 26)	(n = 27)	
**GSRS-IBS: Total Score**			
Baseline (week 0)	27.08 ± 8.32	28.62 ± 9.25	ns
End of intervention (week 8)	26.48 ± 13.08	26.31 ± 14.53	ns
End of follow-up (week 10)	21.92 ± 14.1 *	22.35 ± 14.19 *	ns
**IBS-SSS: Total Score**			
Baseline (week 0)	154.2 ± 72.58	187.88 ± 93	ns
End of intervention (week 8)	140.2 ± 78.14	166.92 ± 93.39	ns
End of follow-up (week 10)	143.4 ± 73.65	163.65 ± 80.41 *	ns
**GIQLI: Total Score**			
Baseline (week 0)	99.32 ± 9.68	99.35 ± 14.69	ns
End of intervention (week 8)	101.68 ± 10.76	101.88 ± 15.28	ns
**VSI: Total Score**			
Baseline (week 0)	54.96 ± 19.04	55.54 ± 19.49	ns
End of intervention (week 8)	60.64 ± 16.65	64.58 ± 15.62 *	ns
**Bowel movements/week**			
Baseline (week 0)	10.52 ± 6.25	8.58 ± 3.68	ns
End of intervention (week 8)	10.48 ± 6.84	8.96 ± 3.8	ns
**Bowel movements/day**			
Baseline (week 0)	1.5 ± 0.89	1.23 ± 0.53	ns
End of intervention (week 8)	1.5 ± 0.98	1.28 ± 0.54	ns

# Change in HT-ES1 vs. placebo, ns: non-significant. Intragroup change from baseline: * *p* < 0.05.

## Data Availability

The data that support the findings of this study are available from the corresponding author upon reasonable request.

## Supplementary Materials

The following supporting information can be downloaded at: https://www.mdpi.com/article/10.3390/nu16223952/s1, Figure S1: Relative abundances of the thirty most abundant genera of all the samples. Figure S2: Correlation plot showing Spearman’s rank correlation coefficient between all clinical variables and organic compound concentrations; Figure S3: Correlations: Heatmaps showing the Maaslin2 Coefficient (Coeff) of correlations between organic compound concentrations and ASVs abundances. Label others includes the rest of the genera and the taxa that could not be annotated at genus level; Table S1: Baseline participant (A) characteristics, (B) habits, and (C) medical history. Table S2: Per-protocol participant characteristics over the intervention. Table S3: Safety parameters over the intervention period. Table S4: Full questionnaire scores. Table S5: PERMANOVA analysis. (***) *p*-value < 0.001. (A) The time, group, the Group:Time interaction the subject and the MEDAS index were included in the model for testing. Only samples with MEDAS index available were included. R²: partial R². F: pseudo-F statistic. (B) The time, group, the Group:Time interaction and the subject were included in the model for testing. R²: partial R². F: pseudo-F statistic. Section S1: Inclusion and exclusion criteria; Section S2: Detailed methods of assessment tools.
