# Supplementary material for: Effect of Postbiotic Bifidobacterium longum CECT 7347 on Gastrointestinal Symptoms, Serum Biochemistry, and Intestinal Microbiota in Healthy Adults: A Randomised, Parallel, Double-Blind, Placebo-Controlled Pilot Study
Source: Nutrients. 2024 Nov 19;16(22):3952. doi: 10.3390/nu16223952 (PMC11597252; doi:10.3390/nu16223952)
Supplement: Supplementary file 1 [file nutrients-16-03952-s001.zip › nutrients-3299848-supplementary.pdf]

nutrients

## Supplementary Materials

### Section S1. Inclusion and exclusion criteria.

Subjects were included if they were aged 18 between 65 years and scored between 13 and 39 points on the GSRS-IBS. Subjects meeting any of the following criteria were excluded from the study: BMI <18.5 or >35 kg/m$^2$, participation in other studies, recent consumption of oral antibiotics or supplements (antioxidants, omega-3, vitamins, minerals, prebiotics, synbiotics or probiotics). Additionally, subjects diagnosed with conditions (dementia, mental illness, cognitive decline, diabetes mellitus type 1 or 2, metabolic syndrome, hypo-hyperthyroidism, autoimmune diseases, eating disorders, serious diseases, or chronic intestinal pathologies) were excluded. Further exclusions included, recent major surgeries, GI surgery within the past 6 months, history of weight loss surgery, recent acute GI illness, allergies to product/placebo, frequent use of antidiarrheal medications, use of corticosteroids, immunosuppressants, or biological drugs in the past 12 months, anticoagulant therapy, alcohol consumption exceeding 30g/day, pregnancy or breastfeeding.

### Section S2: Detailed methods of assessment tools:

The MEDAS questionnaire consists of 14 items that assess adherence to the Mediterranean Diet. Three days food record was used to evaluate MEDAS score by trained personnel. Scores of over 9 points are considered to represent good adherence to the Mediterranean diet. The IPAQ, is a standardized survey designed to measure physical activity levels across different countries and cultural contexts, and it consists of 14 questions all evaluating frequency and intensity of usual physical activity.

The IBS-SSS is a widely used tool for assessing IBS symptom severity. It includes five questions, each scored on a scale from 0 to 100, with the total score ranging between 0 and 500. The questions relate to abdominal pain severity, pain frequency, bloating severity, bowel habits, and interference with quality of life. A score >300 indicates severe IBS symptoms, a score of 175-299 indicates moderate symptoms, and a score of 75-174 indicates mild symptoms.

The GIQLI questionnaire assesses the GI quality of life. It consists of 36 questions and each question is scored on a 5-point Likert scale ranging from 0 to 4 (with higher scores indicating a higher quality of life and a lower score indicating a poorer quality of life). The questions address emotional dysfunction, physical dysfunction, social dysfunction, and satisfaction with medical treatment. The total score ranges from 0 to 144.

The VSI is a validated tool for assessing anxiety specifically related to GI symptoms. It consists of 15 questions, measured on a Likert scale where 1 is completely agree and 6 completely disagree. The total score ranges from 0-90 with a lower score indicating greater anxiety.

The BSS is a visual tool that categorizes stool form into 7 types (from 1 indicating hard lumps to 7 meaning entirely liquid stool) to identify predominance of bowel movements suggesting constipation or diarrhoea. Participants were instructed to choose the form that best described their faecal samples at each bowel movement for 7 days prior to each visit.

**Table S1.** Baseline participant (A) characteristics, (B) habits and (C) medical history.

**Table S1A.** Baseline characteristics of study participants (X±SD)

| | **Population Baseline Values** | **HT ES1 n= 30** | **Placebo n= 30** | **P-value** |
|---|---|---|---|---|
| General | Age (Years) | 43.27 ± 11.56 | 44.3 ± 15.60 | ns |
| | Weight (kg) | 66.51±14.49 | 64.5±10.87 | ns |
| | BMI (kg/m^2) | 24.23±4.31 | 23.78±3.59 | ns |
| | WC (cm) | 81.19±12.84 | 79.76±11.21 | ns |
| Biochemistry | Glucose (mg/dL) | 92.67±6.31 | 93±8.12 | ns |
| | Cholesterol (mg/dL) | 191.83±29.45 | 188.97±29.38 | ns |
| | HDL (mg/dL) | 58.07±12.69 | 63±15.49 | ns |
| | LDL (mg/dL) | 133.67±27.1 | 126±29.63 | ns |
| | Non-HDL (mg/dL) | 155.47±213.66 | 109.07±26.25 | ns |
| | Triglycerides (mg/dL) | 85.43±48.28 | 86.27±40.06 | ns |
| | Total Proteins (g/dL) | 7.14±0.35 | 7.2±0.6 | ns |
| | Albumin (mg/dL) | 4.56±0.21 | 4.55±0.3 | ns |
| | Prealbumin (mg/dL) | 24.67±4.63 | 25.97±3.43 | ns |
| | Creatinine (mg/dL) | 0.74±0.16 | 0.76±0.14 | ns |
| | RTP (mg/dL) | 4.39±1.03 | 4.65±0.87 | ns |
| | CRP (mg/L) | 2.59±2.79 | 1.87±2.27 | ns |
| | ALT (UI/L) | 31.2±32.3 | 24.33±12.82 | ns |
| | Bilirubin (mg/dL) | 0.61±0.15 | 0.71±0.31 | ns |
| | Zonulin (stool) (ng/mg) | 209.15±144.93 | 183.44±129.40 | ns |
| Blood Pressure & Heart Rate | SBP (mmHg) | 107.5±19.4 | 105.1±12.35 | ns |
| | DBP (mmHg) | 70.63±11.7 | 69.07±9.18 | ns |
| | HR (bpm) | 75.67±15.31 | 73.27±9.45 | ns |

HDL: High-Density Lipoprotein, LDL: Low-Density Lipoprotein, SBP: Systolic Blood Pressure, DBP: Diastolic Blood Pressure, HR: Heart Rate, BMI: Body Mass Index, WC: Waist Circumference, RTP: Retinol transporter protein, CRP: C-Reactive Protein, SWMT: Satisfaction with Medical Treatment, DS: Dysfunction Scale. P-values were calculated using independent samples t-tests. ns: non-significant.

**Table S1B.** Baseline participants' habits

| **Habit** | **Percent of population (%)** | **Average Consumption** | **Placebo** | **HT ES1** | **P-value** |
|---|---|---|---|---|---|
| Smoking | 11.7% | 2.1±3.3 cigarettes/day | 2.4±6.6 | 2.0±12.3 | 0.569 |
| Alcohol | 75.0% | 1.3±1.1 times per week | 1.2±0.9 | 1.3±1.2 | 0.884 |

**Table S1C.** Baseline participants' medical history

| **Medical History** | **Percent of population (%)** |
|---|---|
| Risk factors associated with alterations related to glucose metabolism, blood pressure and/or lipid profile | 15.0% |

| | |
|---|---|
| Surgical treatment throughout their lives | 73.3% |
| Had some type of intervention in the last 5 years (varicose veins, meniscus surgery, caesarean section, myopia, etc.) | 26.6% |

**Table S2.** Per protocol participants characteristics over the intervention.

| | **HT-Probiotic n=26** | | **Control n=27** | |
|---|---|---|---|---|
| | **Week 0** | **Week 8** | **Week 0** | **Week 8** |
| **Vitamins and minerals** | | | | |
| Sodium (mmol/L) | 141.31±4.64 | 140±1.74 | 135.59±23.38 | 139.93±1.94 |
| Potassium (mmol/L) | 4.52±0.37 | 4.38±0.3 | 4.41±0.36 | 4.46±0.3 |
| Chlorine (mmol/L) | 105.81±2.19 | 106.08±2.06 | 105.74±1.91 | 106.3±2.54 |
| Calcium (mg/dL) | 9.77±0.31 | 9.73±0.36 | 9.64±0.44 | 9.71±0.31 |
| Phosphate (mg/dL) | 3.47±0.51 | 3.64±0.69 | 3.59±0.59 | 3.73±0.5 |
| Magnesium (mg/dL) | 1.95±0.14 | 1.92±0.24 | 1.99±0.13 | 2.02±0.15 |
| Vitamin B12 (pg/L) | 483.46±150.13 | 464.23±143.25 | 449.44±110.47 | 430.22±114.61 |
| Folate (ng/mL) | 10.31±2.84 | 10.93±3.35 | 10.2±2.92 | 10.39±3.04 |
| Vitamin D (ng/mL) | 19.85±9.20 | 26.81±10.38*** | 19.41±8.42 | 22.89±9.46 |
| Vitamin A (µg/mL) | 0.50±0.12 | 0.52±0.15 | 0.54±0.12 | 0.56±0.10 |
| Vitamin E (µg/mL) | 14.24±2.25 | 14.77±3.72 | 15.17±3.25 | 16±2.87* |
| RBP | 4.43±1.08 | 4.48±1.01 | 4.63±0.88 | 4.85±1.05 |
| **Anthropometrics and body composition** | | | | |
| Weight (kg) | 65.83±13.33 | 66.02±13.71 | 64.85±11.39 | 64.89±11.85 |
| BMI (kg/m^2) | 24±3.83 | 24.15±4.01 | 23.74±3.62 | 23.74±3.75 |
| WC (cm) | 80.58±11.47 | 79.65±11.85 | 79.55±11.55 | 79.99±11.89 |
| Resistance (Ohm) | 603.28±83.37 | 607.93±79.87 | 616.25±57.43 | 620.97±60.87 |
| Reactance (Ohm) | 58.81±6.96 | 58.56±5.68 | 58.57±5.4 | 59.41±5.24 |
| Phase angle deg | 5.62±0.63 | 5.54±0.56 | 5.43±0.55 | 5.47±0.52 |
| Lean mass (kg) | 46.62±9.74 | 46.78±9.3 | 45.08±5.63 | 45.09±5.87 |
| FFMI kg/m$^2$ | 17.71±4.64 | 17.77±4.37 | 17±2.92 | 17.02±3.05 |
| Fat mass (kg) | 18.89±7.42 | 19.89±8.36** | 19.77±7.0 | 20.25±7.43 |
| Fat Mass % | 28.18±7.86 | 28.61±8.97 | 29.79±5.6 | 30.53±6.51 |
| BCM kg | 24.67±5.87 | 24.52±5.67 | 23.37±3.66 | 23.48±3.75 |
| Total Water L | 34.13±7.42 | 34.09±6.84 | 32.99±4.17 | 32.93±4.36 |
| Total Body Water % | 52.17±6.18 | 51.79±6.19 | 51.35±3.99 | 51.28±4.18 |
| Extracellular Water L | 16.2±3.27 | 16.27±2.85 | 15.99±1.84 | 15.85±1.94 |
| Extracellular Water % | 47.69±3.05 | 47.98±2.83 | 48.61±2.78 | 48.26±2.61 |
| Intracellular Water L | 17.93±4.41 | 17.83±4.19 | 17.26±3 | 17.07±2.71 |

Change of HT ES1 vs. placebo: $^{\#}p < 0.05$

Intragroup change from baseline: *p < 0.05, **p < 0.01, ***p < 0.001

Intragroup comparisons over the intervention period were conducted using paired samples t-tests based on variable distribution. Changes from baseline to the end of the intervention (week 8 to week 0) and from week 8 to follow-up (week 10) were analyzed with independent samples t-tests. All tests were two-tailed, with significance set at $p < 0.05$.

BCM: Body Cell Mass. FFMI: Fat Free Mass Index, RBP: Retinol Binding Protein. ns: non-significant.

**Table S3.** Safety parameters over the intervention period.

| | **HT-ES1 n=26** | | **Control n=27** | | |
|---|---|---|---|---|---|
| | **Week 0** | **Week 8** | **Week 0** | **Week 8** | **P-value#** |
| **Kidney function** | | | | | |
| Creatinine (mg/dL) | 0.76±0.15 | 0.77±0.14 | 0.75±0.12 | 0.78±0.1 | ns |
| GFR (mL/min/1.73m$^2$) | 80.6±13.74 | 80.8±5.63 | 82.43±4.43 | 81.18±5.6 | ns |
| **Liver function** | | | | | |
| ALT (UI/L) | 32.35±34.54 | 26.15±12.3 | 22.93±11.09 | 22.96±9.36 | ns |
| Bilirubin (mg/dL) | 0.61±0.15 | 0.67±0.17* | 0.72±0.32 | 0.72±0.19 | ns |
| **Vital signs** | | | | | |
| SBP (mmHg) | 109.81±19.61 | 108.31±17.59 | 106.04±12.6 | 102.56±12.82* | ns |
| DBP (mmHg) | 71.58±11.95 | 72.08±11.87 | 69.59±9.46 | 68.15±9.67 | ns |
| HR (bpm) | 75.73±16.01 | 71.73±12.94 | 73.19±9.1 | 69.74±8.36 | ns |

Change of HT ES1 vs. placebo: #$p < 0.05$

Intragroup change from baseline: *$p < 0.05$, **$p < 0.01$, ***$p < 0.001$

Intragroup comparisons over the intervention period were conducted using paired samples t-tests based on variable distribution. Changes from baseline to the end of the intervention (week 8 to week 0) and from week 8 to follow-up (week 10) were analyzed with independent samples t-tests. All tests were two-tailed, with significance set at $p < 0.05$.

ns: non-significant.

**Table S4.** Full Questionnaire Scores.

| | **HT ES1 (n=26)** | **Placebo (n=27)** |
|---|---|---|
| <u>GSRS-IBS</u> | | |
| **Abdominal Pain Scale** | | |
| Baseline (week 0) | 2.12±0.67 | 2.12±0.86 |
| End of Intervention (week 8) | 2.18±1.09 | 2.38±1.46 |
| End of follow-up (week 10) | 1.74±1.38 | 1.88±1.21 |
| **Bloating Scale** | | |
| Baseline (week 0) | 2.77±0.94 | 3.06±1.07 |
| End of Intervention (week 8) | 2.84±1.32 | 2.97±1.4 |
| End of follow-up (week 10) | 2.11±1.5 ** ~ | 2.58±1.33 |
| **Constipation Scale** | | |
| Baseline (week 0) | 1.44±1.52 | 1.33±1.29 |
| End of Intervention (week 8) | 1.74±1.71 | 1.33±1.61 |
| End of follow-up (week 10) | 1.62±1.36 | 1.06±1.3 |
| **Diarrhea Scale** | | |
| Baseline (week 0) | 2.03±0.65 | 2.2±0.91 |
| End of Intervention (week 8) | 1.78±1.31 | 1.77±1.16 |
| End of follow-up (week 10) | 1.49±1.09** | 1.52±1.13* |
| **Satiety Scale** | | |
| Baseline (week 0) | 1.76±1.51 | 1.87±1.01 |
| End of Intervention (week 8) | 1.5±1.51 | 1.44±1.44 |
| End of follow-up (week 10) | 1.46±1.45 | 1.33±1.46 |
| **Total** | | |
| Baseline (week 0) | 27.08±8.32 | 28.62±9.25 |
| End of Intervention (week 8) | 26.48±13.08 | 26.31±14.53 |
| End of follow-up (week 10) | 21.92±14.1* | 22.35±14.19* |
| <u>IBS-SSS</u> | | |
| Total Score | | |
| Baseline (week 0) | 154.2±72.58 | 187.88±93 |
| End of Intervention (week 8) | 140.2±78.14 | 166.92±93.39 |
| End of follow-up (week 10) | 143.4±73.65 | 163.65±80.41* |
| <u>GIQLI</u> | | |
| **Symptom Scale** | | |
| Baseline (week 0) | 2.68±0.23 | 2.75±0.4 |
| End of Intervention (week 8) | 2.81±0.31* | 2.83±0.38 |
| **Emotional Dysfunction Scale** | | |
| Baseline (week 0) | 2.36±0.43 | 2.19±0.45 |

| | | |
|---|---|---|
| End of Intervention (week 8) | 2.32±0.44 | 2.43±0.52**$ |
| **Physical Dysfunction Scale** | | |
| Baseline (week 0) | 2.74±0.61 | 2.58±0.71 |
| End of Intervention (week 8) | 2.84±0.56 | 2.7±0.78 |
| **Social Dysfunction Scale** | | |
| Baseline (week 0) | 3.48±0.62 | 3.52±0.57 |
| End of Intervention (week 8) | 3.36±0.63 | 3.34±0.66 |
| **Satisfaction with Medical Treatment** | | |
| Baseline (week 0) | 3.56±0.82# | 3.96±0.2# |
| End of Intervention (week 8) | 3.32±0.9 | 3.65±0.69* |
| **Total Score** | | |
| Baseline (week 0) | 99.32±9.68 | 99.35±14.69 |
| End of Intervention (week 8) | 101.68±10.76 | 101.88±15.28 |
| **VSI** | | |
| Total Score | | |
| Baseline (week 0) | 54.96±19.04 | 55.54±19.49 |
| End of Intervention (week 8) | 60.64±16.65 | 64.58±15.62* |
| **Bowel movements** | | |
| **Per week** | | |
| Baseline (week 0) | 10.52±6.25 | 8.58±3.68 |
| End of Intervention (week 8) | 10.48±6.84 | 8.96±3.8 |
| **Per day** | | |
| Baseline (week 0) | 1.5±0.89 | 1.23±0.53 |
| End of Intervention (week 8) | 1.5±0.98 | 1.28±0.54 |

Intragroup change from baseline: *p < 0.05, **p < 0.01, ***p < 0.001

Intragroup change from end of the intervention: ~p < 0.05

Differences between treatments at baseline: #p < 0.05

Change of HT ES1 vs. Placebo: $p = 0.038

Intragroup comparisons over the intervention period were conducted using paired samples t-tests based on variable distribution. Changes from baseline to the end of the intervention (week 8 to week 0) and from week 8 to follow-up (week 10) were analyzed with independent samples t-tests. All tests were two-tailed, with significance set at p < 0.05.

**Table S5.** PERMANOVA analysis. (***) p-value < 0.001. (A) The time, group, the Group:Time interaction the subject and the MEDAS index were included in the model for testing. Only samples with MEDAS index available were included. $R^2$: partial $R^2$. F: pseudo-F statistic. (B) The time, group, the Group:Time interaction and the subject were included in the model for testing. $R^2$: partial $R^2$. F: pseudo-F statistic.

| **(A)** | **Deegres of free-dom** | **SumOfSqs** | **$R^2$** | **F** | **P-value** | |
|---|---|---|---|---|---|---|
| Time | 1 | 0.2032 | 0.0080 | 3.4762 | 0.001 | *** |
| Group | 1 | 0.5149 | 0.0203 | 8.8097 | 0.001 | *** |
| Subject | 49 | 21.7026 | 0.8570 | 7.5787 | 0.001 | *** |
| MEDAS | 1 | 0.0512 | 0.0020 | 0.8758 | 0.732 | - |
| Time: Group | 1 | 0.0478 | 0.0019 | 0.8171 | 0.824 | |
| Residual | 48 | 2.8052 | 0.1108 | | | |
| Total | 101 | 25.3248 | 1 | | | |
| **(B)** | **Deegres of free-dom** | **SumOfSqs** | **$R^2$** | **F** | **P-value** | |
| Time | 2 | 0.3332 | 0.0084 | 2.9667 | 0.001 | *** |
| Group | 1 | 0.7852 | 0.0192 | 13.9818 | 0.001 | *** |
| Subject | 51 | 32.8484 | 0.8253 | 11.4693 | 0.001 | *** |
| Time: Group | 2 | 0.1045 | 0.0026 | 0.9304 | 0.675 | |
| Residual | 102 | 5.7281 | 0.1439 | | | |
| Total | 158 | 39.7994 | 1 | | | |

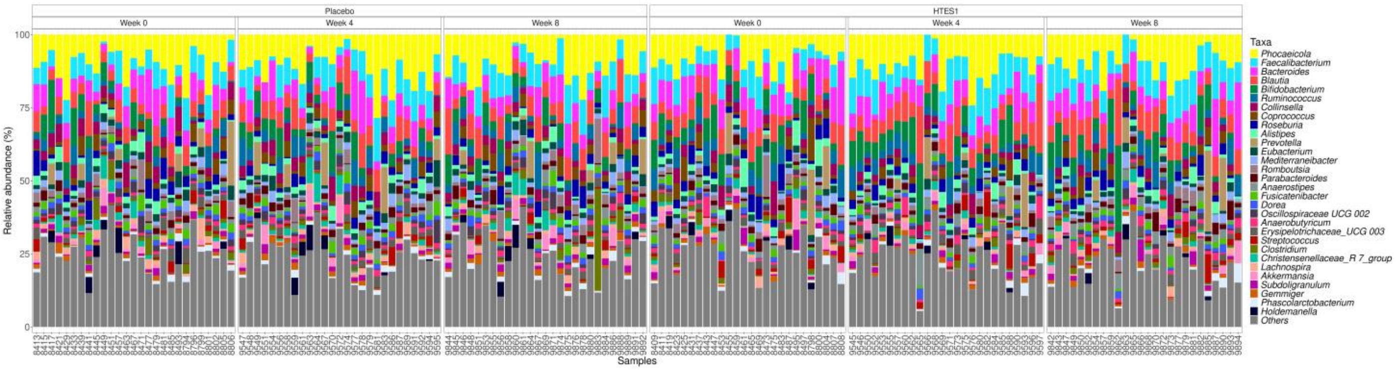


**Figure S1.** Relative abundances of the thirty most abundant genera of all the samples. Label Others includes the rest of the genera and the taxa that could not be annotated at genus level.

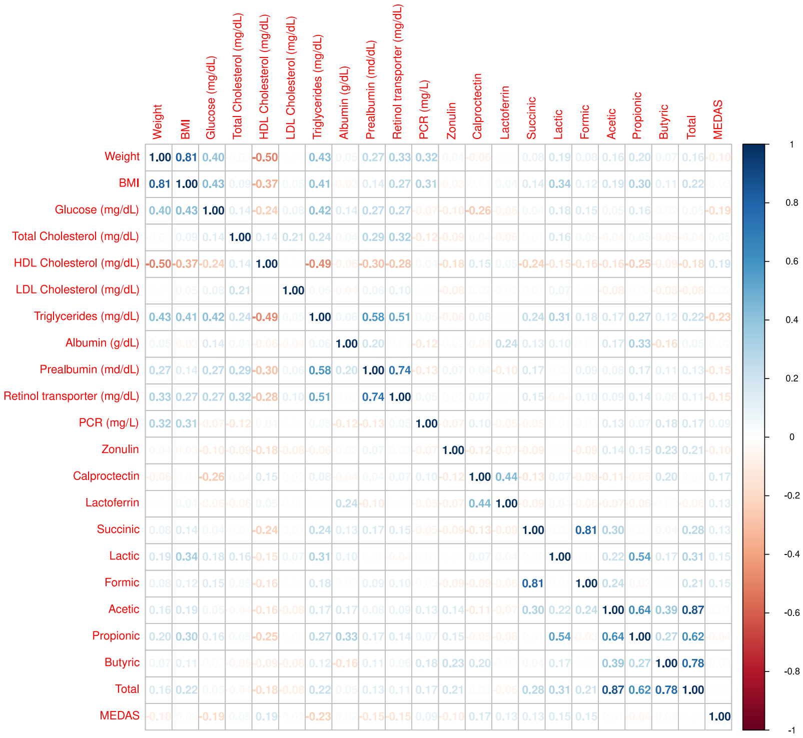

**Figure S2.** Correlation plot showing Spearman's rank correlation coefficient between all clinical variables and organic compounds concentrations.

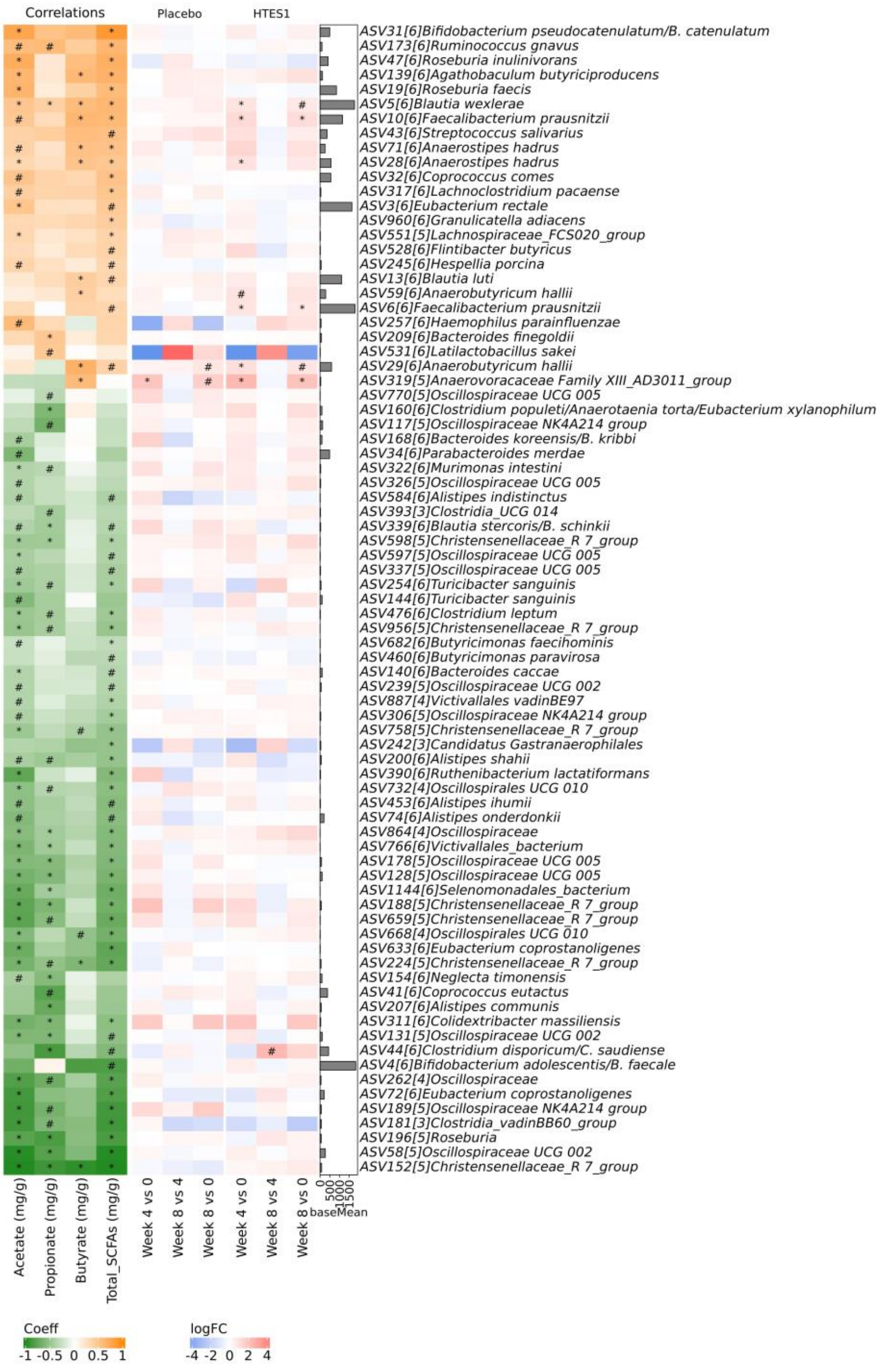


**Figure S3.** Correlations: Heatmaps showing the Maaslin2 Coefficient (Coeff) of correlations between organic compounds concentrations and ASVs abundances. (#): adj-p-value < 0.1. (*) adj p-value <0.05. Orange colour means it is directly correlated. while green colour means inversely correlated. Heatmaps showing the 'log fold change' (Log2FC) resulting from the between times comparisons on ASVs abundances on each group. Red colour means that the taxon is over-represented in the first group of the comparison. while blue colour means that the taxon is over-represented in the second group. (#): adj.p < 0.1. (*): adj.p < 0.05. In (#) and (*) the presence of the taxon in at least 50% samples of at least one of the compared groups. BaseMean barplots show the mean abundances of each ASV.
